# Eviction as a community health exposure

**DOI:** 10.1016/j.socscimed.2023.116496

**Published:** 2023-12-07

**Authors:** Gabriel L. Schwartz, Kathryn M. Leifheit, Mariana C. Arcaya, Danya Keene

**Affiliations:** a Philip R. Lee Institute for Health Policy Studies, University of California San Francisco, San Francisco, CA, USA; b Department of Pediatrics, David Geffen School of Medicine, University of California Los Angeles, Los Angeles, CA, USA; c Department of Urban Studies & Planning, Massachusetts Institute of Technology, Cambridge, MA, USA; d Department of Social & Behavioral Sciences, Yale School of Public Health, New Haven, CT, USA; e Urban Health Collaborative & Department of Health Management and Policy, Drexel University Dornsife School of Public Health, Philadelphia, PA, USA

**Keywords:** Housing, Eviction, Community health, Neighborhoods, Social determinants of health, Social cohesion

## Abstract

Evidence suggests that being evicted harms health. Largely ignored in the existing literature is the possibility that evictions exert *community*-level health effects, affecting evicted individuals’ social networks and shaping broader community conditions.

In this narrative review, we summarize evidence and lay out a theoretical model for eviction as a community health exposure, mediated through four paths: 1) shifting ecologies of infectious disease and health behaviors, 2) disruption of neighborhood social cohesion, 3) strain on social networks, and 4) increasing salience of eviction risk. We describe methods for parsing eviction’s individual and contextual effects and discuss implications for causal inference. We conclude by addressing eviction’s potentially multilevel consequences for policy advocacy and cost-benefit analyses.

## 1. Introduction

Residential eviction is a regular occurrence in low- and middle-income communities in the US. For the past two decades, approximately 1 in 40 rental households (representing nearly 4 million people (Graetz et al., 2023)) have been formally evicted each year (The Eviction Lab, 2018), These annual risks accumulate, such that 1 in 7 children in large cities are formally evicted by the time they reach adolescence (Lundberg and Donnelly, 2019). For children living under 50% of the poverty line, that risk is 1 in 4. True eviction rates are higher still, as informal evictions—although difficult to measure—may outnumber formal evictions by as many as 5 to 1 (e.g., being forced to move via extralegal pressure from a landlord: sudden rent hikes, harassment of tenants, etc.) (Gromis and Desmond, 2021).

Mounting evidence suggests evictions harm health. While the field of eviction epidemiology is still young, studies have linked eviction to mortality (Rojas, 2017), COVID-19 infection (Leifheit et al., 2021a; Sandoval-Olascoaga et al., 2021), injection drug use (Pilarinos et al., 2017; Damon et al., 2019), reduced healthcare access (Chen et al., 2021; Schwartz et al., 2022a), increased acute (but decreased primary) healthcare utilization (Schwartz et al., 2022a; Biederman et al., 2022; Collinson and Reed, 2018), sexually transmitted infections (Kennedy et al., 2017; Niccolai et al., 2019), food insecurity (Leifheit et al., 2020a), birth outcomes (Himmelstein and Desmond, 2021; Khadka et al., 2020; Leifheit et al., 2020b), cognitive development (Schwartz et al., 2022b), and mental health (Desmond, 2016; Desmond and Kimbro, 2015; Leifheit et al., 2021b; Vásquez-Vera et al., 2017).

To date, epidemiology has largely treated eviction as an individual phenomenon: evicted people develop worse health. Yet a sizable portion of this evidence base relies on community-level eviction data to construct exposure measures (Leifheit et al., 2021a, 2021b; Sandoval-Olascoaga et al., 2021; Niccolai et al., 2019; Khadka et al., 2020; Vásquez-Vera et al., 2017), due in part to a lack of individual-level data. Few cohort studies, or even cross-sectional population health surveys, collect formal or informal eviction histories (Leifheit and Schwartz, 2023). In contrast, The Eviction Lab at Princeton University—which operates the only national eviction monitoring effort—makes data on eviction filings and judgements from 2000 to 2018 freely accessible at multiple levels of geographic aggregation (Gromis and Desmond, 2021). Area-level eviction rates thus serve as an attractively available proxy for the eviction risk faced by area residents.

Left largely unexamined is whether area-level eviction rates also represent something more: *contextual* effects local evictions may impose on whole communities. Establishing whether eviction functions as a community-level health risk factor, on top of directly harming the health of evicted people, has real-world implications. First, if the health harms of eviction extend beyond evicted individuals, studies examining the effects of eviction *only on the evicted* would underestimate the full toll of eviction for population health. Second, the design and implementation of governmental policies to prevent evictions (and, importantly, the advocacy case for these policies) may depend heavily on *whose* health is impacted by evictions and how.

In this narrative review, we outline a theoretical model linking area-level evictions and health, using evidence from social science and epidemiology to suggest that area-level evictions represent a cluster of exposures that affect both evicted people and their neighbors. We conclude with implications for research and advocacy.

## 2. A theoretical model for eviction as a community health exposure

**Fig. 1.**
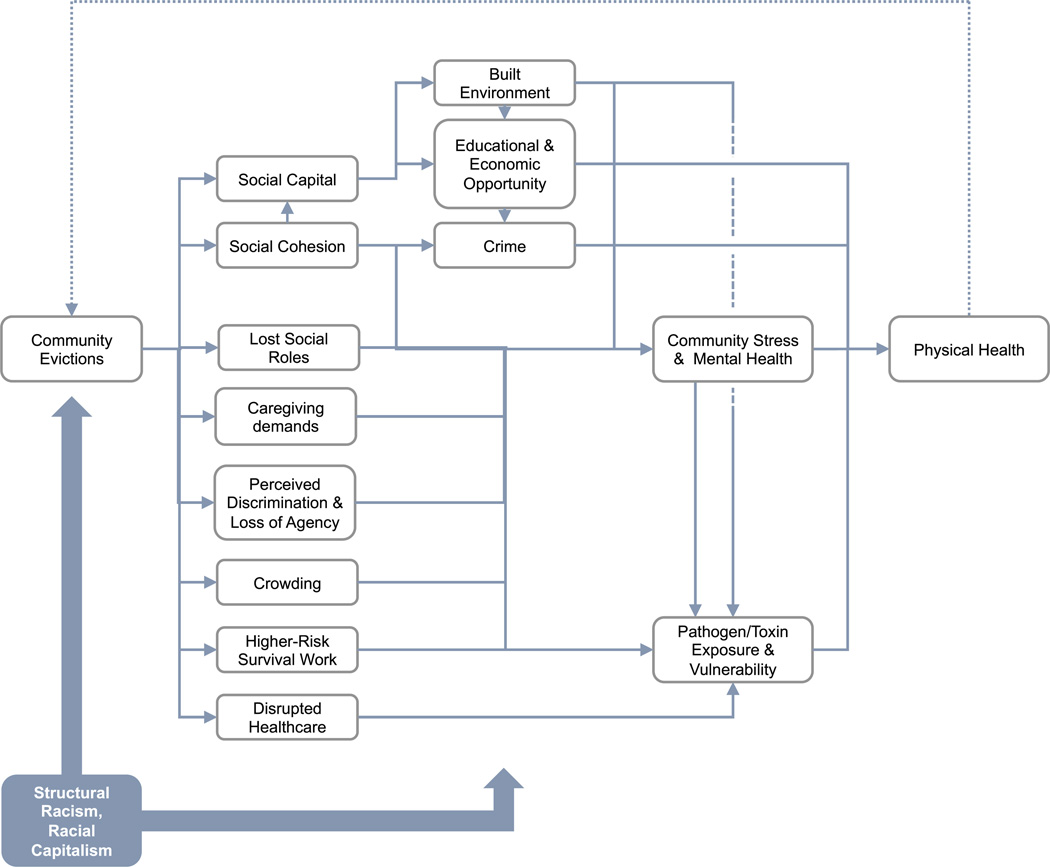
Theoretical model: evictions as a community health exposure Note: This diagram is best read from left to right, starting with community evictions and ending with physical health. Here, structural racism and racial capitalism (A) shape who is at risk of experiencing community evictions as well as (B) modify the relation between community evictions and our theorized mediators, as well as between these mediators and health.

We hypothesize that area-level evictions result in *community-level changes*; these changes then affect the health of non-evicted community members alongside the direct effects of eviction on evicted people. Economists would call such health effects of evictions on non-evicted people ‘spillovers;’ epidemiologists would refer to the phenomenon as ‘interference,’ or among social epidemiologists, ‘contextual effects.’ Although community-level evictions and poor health outcomes share common causes—deeply racialized processes of disinvestment, neglect, and exploitation—evidence points toward a causal link through at least four processes (visualized in Fig. 1): 1) changing ecologies of infectious disease and health behaviors, 2) disruption of neighborhood social cohesion, 3) social network strain, and 4) increasing salience of eviction risk. Though less well-studied, community health may also causally increase community eviction risk, creating a cyclical feedback loop; we discuss this below as an additional, 5th causal path in need of more thorough and direct testing. Throughout, we draw on better-developed models concerning neighborhood foreclosures (Arcaya et al., 2018; Arcaya, 2017; Houle, 2014), as many of the same methodological and theoretical issues apply. We also borrow from epidemiologic work on mass incarceration and police violence, where a community impact lens is better developed (American Public Health Association, 2020; Bor et al., 2018; Jahn et al., 2021; Goin et al., 2021).

### 2.1. Changing ecologies of infectious disease and health behaviors

Where people live and work, and with whom, are elements of local disease ecology, affecting the spread of infectious and vector-borne pathogens. Evictions, in moving people around and clearing out housing units, reshape that ecology.

When evicted families double up with family or friends, residences are more crowded; residents may be unable to socially distance or isolate from one another after an infectious disease exposure. Congregate shelters (a common alternative to living with friends or family) similarly necessitate living in crowded conditions with little personal space (Kendall, 2022; Brown, 2020; Ho, 2020; Baggett et al., 2020; Tobolowsky et al., 2020; Levesque et al., 2022). Simulation studies demonstrate that evictions can thus increase households’ risk of infectious disease—in particular, of contracting and spreading COVID-19 (Nande et al., 2021). Observational studies of meningococcal disease likewise find that adding as few as 2 people to a household *doubles* households’ risk of infection (Baker et al., 2000). Doubling up not only increases risk of disease transmission between household members or residents of congregate shelters; it also increases infectious disease risk for communities, as infected people move through community settings. Individuals may also preemptively double up in order to better afford housing costs and prevent eviction. The threat of eviction alone may thus drive crowding-induced infectious disease transmission even if an individual is not themselves evicted (Arcaya et al., 2020).

Crowding is not the only problem. The threat of eviction causes stress, which can dysregulate the immune system, decreasing the body’s ability to fight an infection (Godbout and Glaser, 2006; Schubert, 2014). Further, eviction threat can change workplace exposures: Arcaya et al. for example, find that low-income renters frequently took on additional front line work during the COVID-19 pandemic in order to make housing payments, opening up a potential pathway between housing displacement pressure and exposure risk (Arcaya et al., 2020). Eviction and related displacement could additionally reshape day-to-day mobility, adding commuting time or necessitating greater mass transit use.

The hypothesis that area-level evictions can drive infectious disease risk is supported by real-world studies leveraging the quasi-random timing of state eviction moratorium expirations during the COVID-19 pandemic. Analyzing data at the state and individual level, these studies estimate that state eviction moratoria prevented hundreds of thousands of COVID-19 cases and tens of thousands of COVID-19 deaths from March to September 2020 alone; effects were particularly large for (though not exclusive to) those living in lower income and more rent-burdened areas (Leifheit et al., 2021a; Sandoval-Olascoaga et al., 2021). Others have found that rising housing costs at the municipal level predict higher COVID-19 case rates, even when accounting for an array of other socioeconomic factors; this association was strongest in communities where residents were disproportionately low-income renters (Arcaya et al., 2020).

Eviction’s impact on infectious disease risk does not appear limited to COVID-19. Additional studies have found strong associations with sexually transmitted infections (Niccolai et al., 2019) and HIV outcomes (Kennedy et al., 2017; Groves et al., 2021). Two interacting mechanisms may mediate these risks, one behavioral and one healthcare-related. First, eviction can change how, when, whether, and under what circumstances people have sex. Eviction may drive the adoption of survival sex work or sexual relationships that exchange sex for housing, contexts where people face economic coercion and thus may be less able to advocate for condoms or limit their number of sexual partners (Groves et al., 2021). Eviction-induced poverty may simultaneously make condoms less affordable, exacerbating barriers to safe sex (Shen et al., 2022). Specific to HIV and other blood-borne infections, eviction is also associated with syringe sharing among youth who use injectable drugs, increasing HIV transmission risk (Pilarinos et al., 2017). This may be driven by eviction-induced possessions loss (including personal injection supplies), displacement away from local syringe exchanges, and increased public drug use.

Second, evictions reduce patients’ healthcare access. In a study examining Medicaid patients in New York City, Schwartz and colleagues (Schwartz et al., 2022a) found that, in the aftermath of an eviction, evicted patients experienced starkly increased risk of Medicaid disenrollment, filled fewer medication prescriptions, and used less ambulatory care. Similarly, literature shows that evictions have deleterious effects on (ability to maintain) adherence to HIV medication regimens and viral load, which are important for reducing community HIV transmission (Kennedy et al., 2017). Sexually transmitted infections may thus become more common not only due to increased or less protected sexual activity but also because infections are less likely to be detected or treated in the aftermath of an eviction.

Taking this a step further, many health behaviors influenced by the behaviors’ of one’s peers could be shaped by community evictions. Qualitative and quantitative work suggests, for example, that eviction shifts whether someone is using drugs as well as the kinds of drugs they are using and their suppliers of drugs (as they are displaced from their neighborhoods and struggle to maintain access to finances and social support) (Damon et al., 2019; McNeil et al., 2021); this would potentially impact overdose risk, not only among evicted people but among those with whom they share drugs (Brooks-Russell et al., 2014; Card and Giuliano, 2013; Ivaniushina and Titkova, 2021; Lakon et al., 2015; Sacerdote, 2014). Accordingly, at least in urban counties, rising eviction is associated with higher rates of accidental drug and alcohol mortality (Bradford and Bradford, 2020).

### 2.2. Disruption of neighborhood social cohesion

People who experience eviction are community members. They have ties to other community members through which material, informational, and emotional support is given and received. Eviction can disrupt these relationships, undermining the social building blocks that enable community well-being. In particular, evictions may erode communities’ social cohesion and reduce their social capital (Kawachi et al., 2014).

#### 2.2.1. Disrupting social cohesion: A breakdown of community and social ties

High rates of eviction cause renter households to churn in and out of low-income neighborhoods. This state of flux and displacement can leave neighbors struggling to form strong and lasting social ties. Eviction-induced displacement thus has the potential not only of eroding specific interpersonal relationships through which social support is passed, but also destroying the larger protective social structure that geographically rooted community provides (Gardner, 2011; Greenbaum et al., 2008). In particular, social cohesion—the connectedness and solidarity that flows through communities with strong social bonds—can be lost. (A recent study from New York State, for example, shows that ZIP codes with higher eviction rates also display lower levels of economic and social connectivity [Weaver, 2023]).

When communities lose social cohesion, they lose an important health-promoting resource, leading to declines in well-being. This phenomenon of broken social ties and concomitant declines in health was famously illustrated in Roseto, Pennsylvania. In the mid-1900s, Roseto was a community of densely socially connected Italian-Americans that demonstrated remarkable cardiovascular mortality advantages over nearby towns populated by other groups of immigrants (Bruhn et al., 1982). Epidemiologic research found this mortality benefit was driven by the “Roseto effect,” i.e. deep and thickly interwoven social ties generating meaning, positive mental health, and lower stress (with attendant benefits for residents’ physiologic function) (Wolf and Bruhn, 1993). The children of Roseto’s residents, however, became increasingly integrated into the US’ dominant culture of atomization, with individuals retreating into nuclear family units and letting the dense social ties that buttressed their parents’ community dissolve. Roseto residents’ mortality advantage, in turn, disappeared (Egolf et al., 1992). Since Roseto, hundreds of studies have linked social cohesion to health, pointing not only to lower stress but also to the ways more socially cohered communities share information, promote access to jobs and educational opportunities, and provide emotional and material support in times of crisis (Kawachi et al., 2014; Berkman, 2000; Oberndorfer et al., 2022).

Qualitative research paints a rich picture of what is lost when geographically-rooted communities are disrupted, helping to ground our hypothesis about the potential impacts of eviction. For example, research has examined the experiences of public housing residents in the wake of public housing demolitions, elucidating the loss of social cohesion associated with these policy-induced “evictions.” Multiple studies point to the way in which longstanding residential communities can foster close relationships among neighbors; prior to relocation, many public-housing residents described their neighbors as “kinfolk” (Stack, 1975), with public-housing residents operating like “one large family” where residents helped each other and “everyone had your back” (Clampet–Lundquist, 2010; Greenbaum, 2008; Manzo et al., 2008). Residents reported that their relationships with neighbors provided a meaningful *social role* that kept them going. In one study, older adults described their communities as places where they were treated as respected elders who were called on to resolve disputes and care for youth (Keene and Ruel, 2013). Upon losing those roles (when their housing projects were destroyed), former public-housing residents described a grief so profound it felt like losing a family member (Keene and Ruel, 2013). This is not to discount the very real problems public housing can pose for residents, but it does illustrate concrete benefits stable residential communities provide.

Evictions that destroy geographically rooted community, then—particularly mass waves of eviction affecting large portions of neighborhoods’ residents, as regularly occurs in some US cities (Wolf and Bruhn, 1993; Waldman, 2022; Woolley et al., 2008; Bjella, 2022)—may induce grief and depression, which has a well-established influence on physical well-being (Cassano and Fava, 2002; Kubzansky et al., 2014; Penninx et al., 2013; Stroebe et al., 2007; Seiler et al., 2020). Once displaced, public housing demolition studies suggest evicted families may struggle to rebuild their social networks in their new neighborhoods, causing a net negative loss in geographically-rooted social ties (Greenbaum et al., 2008; Clampet- and Lundquist, 2004).

#### 2.2.2. Implications for social capital and political power

Lost social cohesion can also chip away at social capital, defined as the reciprocity, mutual aid, and material resources that flow through socially cohered networks (Kawachi et al., 2014). Reduced social capital renders communities less able to marshal collective power to meet neighborhood needs by pooling community resources (Baylis et al., 2013; Aida, 2018; Flora, 1998) or pressuring local government to invest in their communities (Payne and Williams, 2008; Altschuler et al., 2004; Krishna, 2002). As a consequence, communities may have less control over environmental exposures (e.g., the placement of a highway or pollution source), investments in green space, maintenance of roads and sidewalks, and the availability of economic and educational opportunity (Woolley et al., 2008; Smiley, 2020; Mazumdar et al., 2018).

Accordingly, community evictions are associated with measurable reductions in social capital and political power. At the neighborhood level, rising eviction rates coincide with fewer 311 calls (requests to local government to make basic repairs, such as filling potholes and replacing street signs) (van Holm and Monaghan, 2021) and lower voter turnout (Weaver, 2023; Slee and Desmond, 2023). Related research on “urban renewal,” which led to mass evictions in urban communities of color (particularly, Black communities), similarly shows that forced displacement can lead to declines in political power and subsequent harms to community well-being (Fullilove, 2001; Fullilove and Wallace, 2011). And qualitative research with people displaced by public-housing demolitions points to decreases in civic engagement and collective political agency (Keene, 2016). Across an array of contexts, then, displacement-induced reductions in civic engagement and political capacity hobble communities’ ability to advocate for and create health-promoting structures or curb neighborhood stressors.

#### 2.2.3. Implications for crime victimization

Finally, a breakdown of social cohesion adversely impacts communities’ safety. Absent social cohesion, communities with high rates of eviction may be less socially invested in one another, less likely to interact regularly in public, and less able to provide “eyes on the street” and social accountability to prevent interpersonal violence (Kawachi et al., 2014; Jacobs, 1961). Moreover, lacking the material and emotional support social cohesion fosters, community members may be more likely to engage in crime as a means of survival (Mills and Zhang, 2014) or more likely to experience emotional crises (Kingsbury et al., 2020; Breedvelt et al., 2022; Gullett et al., 2022) that could lead to physical altercations. Increasing neighborhood eviction rates are consequently associated with increases in neighborhood crime (robberies, burglaries, and homicides), (Semenza et al., 2022) a pattern reflected in similar research on foreclosures (Immergluck and Smith, 2006).

### 2.3. Strain on social networks

Evicted people frequently “double up” in the months after an eviction by moving in with friends or relatives (Link et al., 1994). (Though some evicted individuals may move to congregate or emergency shelters, these locations are often in short supply (Moses, 2019) and are typically places of last resort [Robinson et al., 2022; Zeger, 2021]). Qualitative research demonstrates that this doubling up strains social relationships, stressing both evicted people and their new housemates (Desmond, 2016; Babajide et al., 2016; Keene et al., 2022). The combining households must crowd into limited space and navigate potentially conflicting preferences around household rules, schedules, eating habits, etc. Many doubled-up evictees express depression, frustration, and a loss of autonomy (Babajide et al., 2016; Skobba and Goetz, 2015). Focusing on the receiving households, Keene and colleagues find that these “informal housing providers” report multiple stressors associated with providing housing, including crowded conditions, lack of privacy, increase in expenses, caretaking demands, and, importantly, threats to their own housing stability when they violate the terms of their lease in order to take in a network member (Keene et al., 2022). This stress and interpersonal strain can have negative consequences for their health, disrupting sleep and health routines, and straining social bonds that provide health-promoting social support (Desmond, 2016; Keene et al., 2022; Skobba and Goetz, 2015).

While likely felt most acutely among the people evictees move in with, eviction can strain social bonds widely. Low-income communities provide tremendous amounts of care for each other (Stack, 1975). Eviction increases the need for this care, perhaps to a level that is unsustainable; this includes eviction’s capacity to worsen poverty and contribute to acute health crises, criminal legal system involvement, and changes in substance use patterns (Schwartz et al., 2022a; Collinson and Reed, 2018; Desmond, 2016; McNeil et al., 2021; Gottlieb and Moose, 2018). Resulting increased care burdens can directly impact caregivers’ health, including via increased stress, lost sleep, missed work, and more (Bauer and Sousa-Poza, 2015; Ho et al., 2009; Burton and Bromell, 2010). Further, ethnographic work describes interpersonal tension induced when housing-insecure families threatened by eviction repeatedly reach out to their limited network asking for money or a place to live to avoid homelessness (Desmond, 2016). Over time, these repeated asks made many relationships feel untenably transactional and extractive, leading to relationship loss. Eviction can thus serve as an enduring toxin to the social bonds that sustain communities’ health, engendering stress and poor mental health throughout.

### 2.4. Increasing salience of eviction risk

#### 2.4.1. Community evictions as a threat to personal housing security

Area-level evictions may also change residents’ perceptions of their personal eviction risk or the quality of their neighborhoods, with implications for health. Seeing neighbors forced out of their homes and their possessions piled on the street could increase the salience of eviction’s consequences and increase the psychological duress people feel when struggling to pay rent. Evictions are concentrated geographically, with certain buildings and neighborhoods representing an outsize share of metros’ eviction rates (Rutan and Desmond, 2021; Seymour and Akers, 2021a, 2021b; Rudolph et al., 2021). Moreover, “serial eviction” is a well-documented phenomenon, with large landlords batch filing evictions (often repeatedly targeting the same tenant) as a rent collection tool (Immergluck et al., 2020; Leung et al., 2023). These practices could signal to nearby renters that landlords are ready to serve an eviction notices if renters fall behind. This matters for health because the threat of eviction is itself a potent stressor (Leifheit et al., 2021b; Vásquez-Vera et al., 2017). Ethnographic work, for example, demonstrates that poor mental health and psychological stress increase well before an eviction is executed—even before it is filed—suggesting the threat alone is enough to damage health (Desmond, 2016). Data from the COVID-19 pandemic using quasi-experimental methods yield a similar conclusion, showing that state eviction moratoria were effective in reducing population mental distress—but only if they blocked landlords from threatening tenants with eviction via notice and filing (as opposed to merely blocking eviction executions) (Leifheit et al., 2021b).

Papers by Arcaya et al. corroborate the ability of local housing market conditions to affect community residents’ health via spillovers. The authors linked Massachusetts foreclosures to the Framingham Offspring Cohort, regressing health onto the number of recent foreclosures within 100 m of participants’ homes. Even though participants were not themselves foreclosed upon, nearby foreclosures were associated with longitudinal changes to stress-related risk factors for cardiometabolic disease, including increased systolic blood pressure (Arcaya et al., 2014) and weight gain (Arcaya et al., 2013). Effects were partially mediated by alcohol consumption, a common coping behavior in the face of stress. Though the salience of proximate foreclosures and evictions likely differs between homeowners and renters, these studies show that the housing experiences of one household can spill over to affect the stress-related health behaviors and disease risk of their neighbors.

Although less well-documented, hearing about neighbors being evicted might prevent tenants from advocating for their right to safe, health-promoting housing. Fearing eviction, tenants might feel disempowered when it comes time to ask landlords for needed repairs, pest and mold remediation, etc. Such fears are not unfounded: although retaliatory evictions are illegal in most states, tenants often report receiving eviction notices following repair requests (Desmond, 2016; Volk and Christie, 2019; Handa, 2016; Urban et al., 2019). Reporting from Philadelphia, for example, found that tenants who filed complaints about housing conditions frequently received eviction filings within months of the complaint (Volk and Christie, 2019). Though nationwide surveys on tenants’ fear of retaliatory evictions are scarce in the United States, surveys in England (Levelling Up Housing and Communities Committee, 2016) and Australia (CHOICE National Shelter, 2017) find roughly half of each countries’ renters fear their landlord would attempt to evict or “blacklist” them if they requested needed repairs. Seeing neighbors evicted may amplify those fears, driving up the prevalence of habitability violations such as mold, rodent or cockroach infestations or extreme temperatures and increasing tenants’ risk of respiratory illness, injury, and poor mental health (American Lung Association, 2020; Evans et al., 2000; Gielen et al., 2015; Wimalasena et al., 2021).

#### 2.4.2. When communities are targets: structural racism in eviction risk and spatial stigma

Eviction risk is not random. By design—that is, as determined by decades of ongoing interpersonal and structural racism in housing, education, employment, criminal-legal systems, and financial institutions’ assessments of neighborhood and individual investment risk (Swope and Hernández, 2019; Rothstein, 2017; Blankenship et al., 2023; Leifheit et al., 2022)—eviction rates are higher in racially/ethnically marginalized communities. Nationally, Black people represent 13.6% of the population but 18.6% of renters and 43.4% of all evictions; that translates to 1 in 5 Black renters in the US being threatened with eviction each year, roughly half of whom are evicted (Graetz et al., 2023). Indeed, evictions are part of an ongoing legacy of targeted, serial displacement of racially marginalized communities through processes such as urban renewal and gentrification (Fullilove and Wallace, 2011; Rucks-Ahidiana, 2022; Whittaker et al.). Evictions can be wielded as a tool to accelerate racialized dispossession, paving the way for rent increases and a growing share of higher income and White residents (Whittaker et al.; Dantzler, 2021; Moskowitz, 2017). Conversely, prevalent area-level evictions can reify racialized spatial stigma that devalues property, reduces investments, and contributes to psychosocial stress and poor health among residents (Keene and Padilla, 2014; Keene et al., 2018) (while still allowing landlords to extract the highest possible profits [Desmond and Wilmers, 2019]).

Area-level evictions may thus have distinct impacts in racially/ethnically marginalized communities. First, in these communities, housing market activity that displaces neighbors and changes neighborhood conditions, such as frequent local evictions, may be rightfully interpreted as a form of structural racism (Binet et al., 2022). Structural racism in the housing market imposes a psychological cost on residents beyond fear and stress about one’s personal housing situation. A community-based participatory research project focused on gentrification in Eastern Massachusetts, for example, found that higher levels of perceived ownership of neighborhood change was associated with better mental health, even when accounting for individual residential stability (Binet et al., 2022). This research dovetails with a thoughtful literature on (A) sense of control over one’s life and living conditions as a determinant of health (Whitehead et al., 2016), and (B) the psychological impact of understanding one’s neighborhood or school as a target of discrimination, with attendant spatial stigma that affects residents’ sense of self and societal value (Keene et al., 2018; Seaton and Yip, 2009). These matter for health both because having *actually* low power over one’s surroundings and living conditions negatively impacts one’s access to resources and freedom from toxic exposures (see “Implications for social capital and political power*”* above), and because *perceived* low power over one’s surroundings and living conditions induces chronic psychological duress (Whitehead et al., 2016).

Second, the loss of social support and social capital neighborhood evictions engender may have heightened consequences for Black communities given the critical role that identity-affirming social support networks play in mitigating the health impacts of racism (Stack, 1975; Geronimus, 2000). That is: if community institutions and social ties play a uniquely critical role in helping Black communities thrive and survive in the face of structural oppression—not to mention resist and contest it (Michener, 2023)—disrupting those ties and institutions through eviction will have disproportionate consequences for Black communities’ well-being.

### 2.5. Reverse causation

In the opposite direction, individuals’ poor health can increase community risk of evictions. Multiple studies have identified health status and healthcare access as causes of eviction (Schwartz et al., 2021; Allen et al., 2019; Linde and Egede, 2023; Zewde et al., 2019). Previously, we’ve explained these associations as resulting from individual-level processes whereby out-of-pocket costs and disability lead to missed work, financial hardship, and ultimately delinquent rent (Schwartz et al., 2021). But it’s equally true that an individual’s illness and associated costs can strain the social and financial resources of their networks (especially their families [Golics et al., 2013; Skufca and Rainville, 2021]), causing eviction risk spillovers. Caregivers of cancer patients, for example, often miss work, or even lose their jobs due to caregiving responsibilities (Bradley, 2019). Informal caregivers of dementia patients face similar challenges (Cancino and Zinin, 2016).

Eviction and poor health may therefore operate as a feedback loop at the community-level, cyclically accumulating sociobiological disadvantage within geographic and social networks (Fig. 1) (Schwartz et al., 2021). On the one hand, this means eviction and poor health may powerfully (re)produce social stratification, entrapping communities in subjugated sociospatial positions. On the other, intervening to improve community health *or* prevent evictions would intervene *countercyclically*: preserving housing instability would protect communities’ health, which would safeguard communities’ housing, and so on.

## 3. Discussion

The evidence above suggests eviction operates at multiple levels, acting as an individual and contextual health exposure. Researchers must attend to these multilevel dynamics, both because they pose important methodological challenges but also because they have the potential to change our conception of who is treated by eviction (and therefore *whose health would improve* if policymakers lowered eviction rates).

If eviction exerts contextual effects, population attributable risks of eviction would have to be calculated by summing *two* mathematical products: those found by multiplying (1) the impact of individual-level eviction by the number of people who are evicted, and (2) the impact of community-level eviction by the number of people who live in community with evictees. Even a small contextual effect would translate into substantial public health benefits for eviction prevention, given the much larger pool of people we would consider ‘treated.’ A corollary is that individual-level effects are likely to be underestimated in studies examining individual-level evictions alone while ignoring contextual effects; this is because un-evicted sample members may be treated by eviction, too (via its contextual effects), complicating individual-level causal contrasts. As evidence about area evictions as a community health exposure accumulates, epidemiologists must respond by adapting the way we estimate the costs and benefits of different housing policy choices.

Taking the potentially contextual effects of eviction seriously also shifts the constituency for eviction prevention. Eviction of course primarily impacts low- and middle-income households and their communities, and as a result of structural racism hugely disproportionately burdens communities of color (Graetz et al., 2023). But the evidence reviewed above suggests the health harms of eviction may nonetheless stretch beyond evicted families and cross the boundaries of social and geographic segregation (especially for infectious disease) (Sandoval-Olascoaga et al., 2021; Nande et al., 2021). If everyone’s health is put at risk by a neighborhood eviction (however unevenly distributed that risk might be), the well-being of entire communities—and constituencies—is at stake. Expanding who we understand to be impacted by eviction may shape support for eviction prevention policies.

### 3.1. Paths forward: interpreting & estimating area-level eviction rate coefficients

When we see an association between area-level eviction rates and health outcomes, it is hard to know the extent to which the association is driven by effects among evicted households versus community spill-overs. Whether that distinction matters depends on one’s goal. If estimates are used to inform upstream interventions—i.e., some action that prevents eviction at scale—it may not be necessary for an ecological study to disentangle the paths through which eviction is causing poor health. Lowering eviction rates in an area will intervene on all paths between eviction and health, and so the collective impact of area-level evictions on area-level health through all mediating paths is in fact what one wants to estimate. Here, the challenge will be making a compelling causal argument that a given study design has eliminated selection and confounding. Leveraging policy changes as natural experiments is one promising path forward.

If, however, one wanted to: (A) calculate an unbiased estimate of the effects of eviction on evicted individuals; (B) inform downstream interventions—e.g., accurately describing the effects of eviction on individuals’ healthcare access to healthcare payers, thus demonstrating the importance of providing housing supports to the patients they insure; or (C) inform advocacy efforts more broadly, parsing would become consequential, necessitating studies capable of separating out individual from contextual paths. To start, qualitative work exploring the ways eviction shapes the well-being of evicted people’s neighbors and families is essential for fleshing out and testing the pathways described above.

Quantitative studies interested in separating eviction’s individual and contextual effects might use individual eviction and health data measured longitudinally and linked to area-level eviction rates. Say, for example, that we were interested in the relationship between eviction and risk of preterm birth. In a longitudinal cohort, we could collect detailed residential histories on each person over the entire course of their pregnancy; each individual may have moved or been displaced several times, and thus may have lived in anywhere from 1 to n neighborhoods indexed by k. We could then fit the following model:

(1) $$\text{logit}\left(p\right)=\beta_{0}+\beta_{1}Evict+\beta_{2}\sum_{k=1}^{n}\left(\omega_{k}*AreaEvict_{k}\right)+L^{\prime }X$$

where *Evict* represents an indicator for whether each individual was evicted at some point during their pregnancy (or a count of evictions they experienced); $AreaEvict_{k}$ measures the eviction rate in each neighborhood k that a given cohort member lived in during their pregnancy; $\omega_{k}$ is a weight representing the proportion of each person’s pregnancy that they lived in neighborhood k; X is a vector of measured covariates; L is a vector of covariate coefficients; and p is the probability of preterm birth. In this model, the coefficient $\beta_{2}$ would provide an estimate of the contextual effect of evictions, independent of individuals’ direct exposure to eviction.

Such an analysis, of course, requires temporally and spatially detailed residential histories. This is important in general when studying the effects of contextual variables: low income people move frequently, often between neighborhoods that are substantively different with respect to, for example, levels of neighborhood poverty (Jackson and Mare, 2007). For evicted people—100% of whom move, and among whom residential addresses may change rapidly in the wake of their eviction—such detailed histories are especially essential. Using time-fixed contextual measures would obscure this important within-person variation. Ideally, eviction rates would also be measured at fine levels of time and geography, though this is currently rarely available in the US.

In Equation (1), we have assumed that no sample members share neighborhoods. In some cohort studies, such as the Panel Study of Income Dynamics—where respondents are spread out across the entire United States—such an assumption may basically hold. In many multilevel datasets, however, sample members are intentionally selected such that they are collectively representative of the larger units from which they are sampled, e.g., neighborhoods (Sampson et al., 2022). In these cases, neighborhood clustering will loom large. Appropriate models will have to treat individuals as having been nested within, and cross-classified between, residential neighborhoods as they move over time.

### 3.2. Complications for causal inference

The fact that sample members in multilevel datasets may share neighborhoods, however, creates additional problems. In particular, it violates the stable unit treatment value assumption (SUTVA) that the potential outcomes for any sample member i are independent of the treatment assigned to any other sample member j (i.e., the assumption of non-interference, or of no spillovers) (Schwartz et al., 2012). If, as we propose above, member i’s eviction changes the community eviction rate experienced by sample member j, and the effect of community evictions is anything but 0, SUTVA cannot hold.

In the presence of a SUTVA violation, we must apply special methods to estimate average treatment effects (e.g., see Laffers and Mellace [Laffers and Mellace, 2020]). Absent these methods, any study of individual-level evictions in which sample members share neighborhoods can only be said to causally identify the effects of eviction on health if $\beta_{2}$ from Equation (1) above equals 0 (i.e., the contextual effects of eviction are negligible). To estimate how large the bias is when estimating the effects of eviction while ignoring SUTVA violations, simulation studies at various geographic scales would be needed.

The appropriate geographic scale for a given analysis is not obvious. Although we have focused on “neighborhoods” for illustrative purposes, the practical challenge for researchers is to work through (theoretically and empirically) which geographic scales are most relevant for people’s social or ecological networks, broadly defined. Alternately, one could abandon geography entirely and focus on family or kin networks as a unit of analysis; but defining a social network, too, requires scale choices.

Regarding methods for disentangling our hypothesized pathways, one could imagine mediation analyses to estimate each pathway’s relative contribution (Rudolph et al., 2021). It will be difficult, however, to collate data that sufficiently represent (A) each construct along with (B) all exposure-mediator and mediator-outcome confounders. It may be more feasible to build evidence for these paths one by one, finding strong causal designs to test relationships between eviction and each mediator and then between each mediator and health. Such analyses could triangulate across many different methods, including qualitative studies.

## 4. Conclusion

Eviction epidemiology is nascent. Nearly any study rigorously evaluating the impact of individual- or community-level evictions on health is an important step forward for the field. If this literature is to be used to design interventions, as inputs for cost-benefit analyses, or as the basis for advocacy, however, epidemiologists must engage with the likely reality that the health impacts of evictions are not confined to those who are evicted. Conceptualizing evictions as a community-level health exposure requires us to break new analytic and theoretical ground, for the health of evicted families and, inevitably, the health of us all.

## Data Availability

No data was used for the research described in the article.
